# Does Interaction between the Motor and Regulatory Domains of the Myosin Head Occur during ATPase Cycle? Evidence from Thermal Unfolding Studies on Myosin Subfragment 1

**DOI:** 10.1371/journal.pone.0137517

**Published:** 2015-09-10

**Authors:** Daria S. Logvinova, Denis I. Markov, Olga P. Nikolaeva, Nikolai N. Sluchanko, Dmitry S. Ushakov, Dmitrii I. Levitsky

**Affiliations:** 1 A.N. Bach Institute of Biochemistry, Russian Academy of Sciences, Moscow, Russia; 2 Department of Biochemistry, School of Biology, Moscow State University, Moscow, Russia; 3 Department of Biotechnology, School of Biology, Vyatka State University, Kirov, Russia; 4 A.N. Belozersky Institute of Physico-Chemical Biology, Moscow State University, Moscow, Russia; 5 King's College London, London, United Kingdom; Institute of Enzymology of the Hungarian Academy of Science, HUNGARY

## Abstract

Myosin head (myosin subfragment 1, S1) consists of two major structural domains, the motor (or catalytic) domain and the regulatory domain. Functioning of the myosin head as a molecular motor is believed to involve a rotation of the regulatory domain (lever arm) relative to the motor domain during the ATPase cycle. According to predictions, this rotation can be accompanied by an interaction between the motor domain and the C-terminus of the essential light chain (ELC) associated with the regulatory domain. To check this assumption, we applied differential scanning calorimetry (DSC) combined with temperature dependences of fluorescence to study changes in thermal unfolding and the domain structure of S1, which occur upon formation of the ternary complexes S1-ADP-AlF_4_^-^ and S1-ADP-BeF_x_ that mimic S1 ATPase intermediate states S1**-ADP-P_i_ and S1*-ATP, respectively. To identify the thermal transitions on the DSC profiles (i.e. to assign them to the structural domains of S1), we compared the DSC data with temperature-induced changes in fluorescence of either tryptophan residues, located only in the motor domain, or recombinant ELC mutants (light chain 1 isoform), which were first fluorescently labeled at different positions in their C-terminal half and then introduced into the S1 regulatory domain. We show that formation of the ternary complexes S1-ADP-AlF_4_^-^ and S1-ADP-BeF_x_ significantly stabilizes not only the motor domain, but also the regulatory domain of the S1 molecule implying interdomain interaction via ELC. This is consistent with the previously proposed concepts and also adds some new interesting details to the molecular mechanism of the myosin ATPase cycle.

## Introduction

The molecular mechanism of muscle contraction and many other events of biological motility is based on cyclic interaction of myosin heads with actin filaments coupled to myosin–catalyzed ATP hydrolysis. The myosin head which is referred to as myosin subfragment 1 (S1) consists of two major structural domains, the N-terminal globular motor (or catalytic) domain and the C-terminal regulatory domain. The motor domain is responsible for ATP hydrolysis and actin binding. The regulatory domain is a long α-helix stabilized by non-covalent interactions with the essential and the regulatory light chains (ELC and RLC, respectively) [1]. The two domains are connected by one of the subdomains in the motor domain, which is called the ‘‘converter”, that immediately adjoins the regulatory domain and includes the interface between the motor domain and ELC [2].

The present concept of the myosin motor function includes rotation of the regulatory domain relative to the motor domain. During this rotation the regulatory domain acts as a semi-rigid “lever arm”, which amplifies and transmits conformational changes occurring in the motor domain during ATP hydrolysis. Local conformational changes in the myosin ATPase site spread to the entire motor domain, resulting in global structural changes in the motor domain and leading to rotation of the converter by ~60° [3, 4]. The movements of the converter are directly coupled to and amplified by the extended lever arm, which rotates with a hinge point located in the so-called pliant region between the converter and the ELC associated with the regulatory domain [3].

The lever arm concept is supported by the fact that the sliding velocity of actin filaments in the *in vitro* motility assay strongly depends on the length of the lever arm [5]. This concept is also supported by the data showing that destabilizing of the regulatory domain by removal of ELC or RLC (and especially of both) markedly reduces the sliding velocity of actin filaments without a significant loss in actin-activated ATPase activity of myosin [6]. Importantly, it has also been shown by single actin filament force measurements that RLC removal has almost no effect on isometric force, whereas ELC removal reduces the myosin force production by over 50% [7]. These results suggest that ELC associated with the regulatory domain may play a crucial role in the motor function of the myosin head, taking part in the overall stabilization of the S1 molecule during the ATPase cycle.

**Fig 1:**
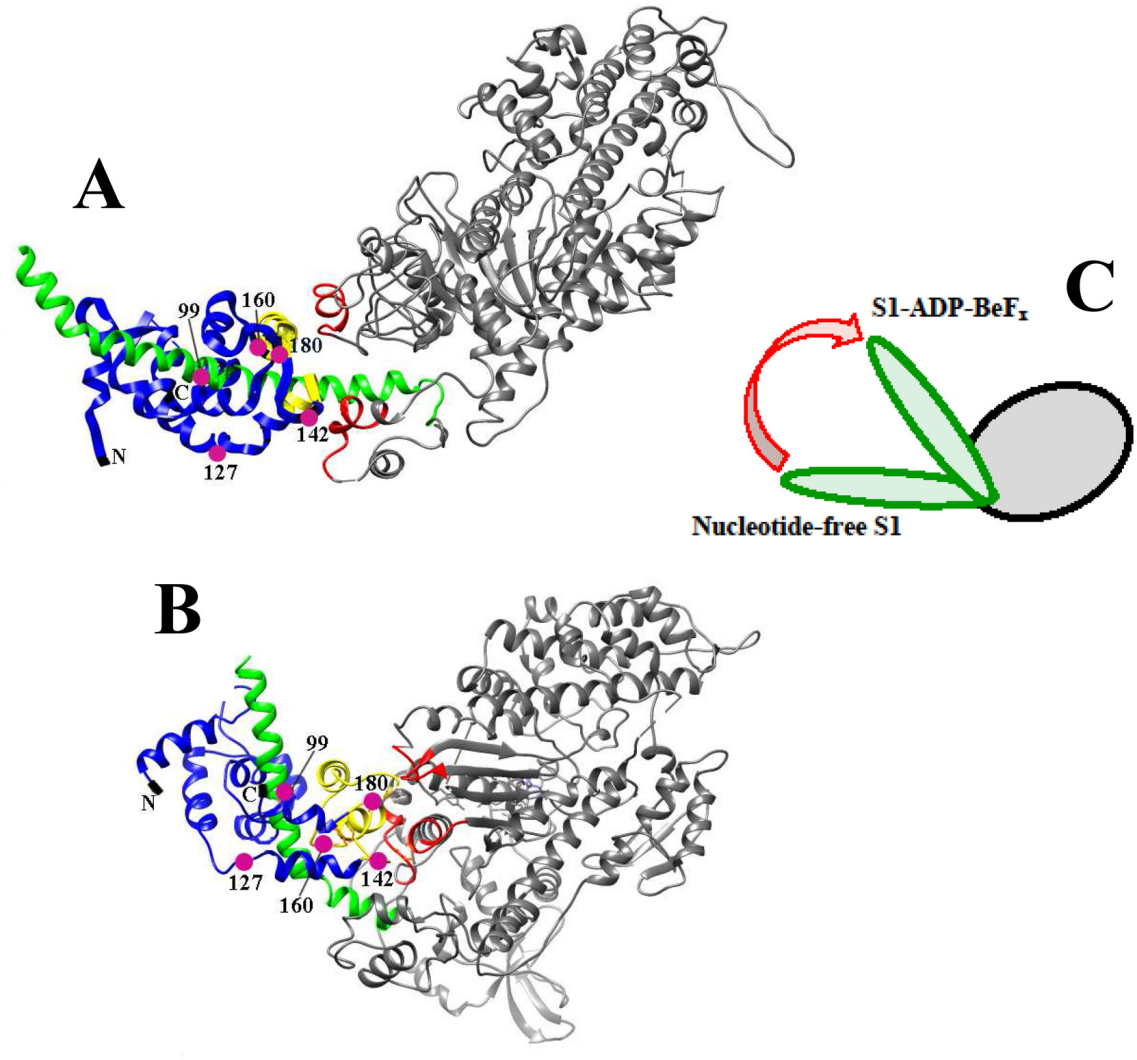
Proposed interactions between the C-terminal half of ELC and the motor domain of the myosin head. Structures of nucleotide-free chicken skeletal S1 (PDB entry 2MYS) **(A)** and chicken gizzard S1 in the S1-ADP-BeF_*x*_ complex (PDB entry 1BR4) **(B)**. The motor domain is grey, the heavy chain of the regulatory domain is green, ELC is blue, and the region in the ELC C-terminal part, which is proposed to interact with the motor domain, is highlighted in yellow, while the regions of the motor domain interacting with this ELC region are highlighted in red. Pink circles show the locations of Cys-180 of the wild-type ELC and the Cys residues in ELC constructs generated by introducing Cys-99, Cys-127, Cys-142, or Cys-160 after removal of Cys-180 by replacing it with Ala (numbers of Cys residues refer to the human ventricular ELC1 [13]). The C-terminal part of the regulatory domain with associated RLC is not shown because it is cleaved off in chymotryptic S1 used in the work. **(C)** Schematic representation of rotation of the S1 regulatory domain (green) relative to the motor domain (grey) upon formation of the S1-ADP-BeF_*x*_ complex.

Atomic structures of the myosin head indicate that rotation of the regulatory domain during the ATPase cycle can be accompanied by interactions of the C-terminal part of ELC with different sites of the motor domain. In the nucleotide-free skeletal muscle S1 structure this part of ELC is located close to the N-terminal part of the heavy chain in the motor domain [1, 8]. In contrast, in the structure of smooth muscle S1 with MgADP-AlF_4_ or MgADP-BeF_x_ bound in the active site (i.e. stable analogs of the S1**-ADP-P_i_ and S1*-ATP intermediate states of the S1-catalyzed Mg^2+^-ATPase reaction) the ELC has swung to the other side of the motor domain where its C-terminal part interacts with a flexible loop at the entrance to the nucleotide-binding cleft [9] (Fig 1). It seems likely that these interactions between ELC and the motor domain, which probably occur during ATPase cycle, may play an important role in the functioning of the myosin head as a molecular motor. In favor of this assumption are the data obtained with myosin S1 containing ELC fluorescently labeled at a single Cys residue (Cys-180) near the C-terminus, which showed that the addition of MgATP to S1 leads to a 6–10% decrease in the fluorescence of the ELC-bound label [10] and this decrease is mainly caused by the ATP hydrolysis step [11]. The concept of rather tight nucleotide-induced interaction between the C-terminal part of ELC and the motor domain of the myosin head is also supported by the results of fluorescence polarization studies on S1 and muscle fibers showing that binding of ATP to the active site on the motor domain has a significant effect on the mobility of the C-terminal part of ELC, which is mobile and partially disordered in the absence of nucleotides, but becomes immobilized and ordered in the presence of ATP [12]. This is partly in line with the results of fluorescence lifetime imaging microscopy studies on skeletal muscle fibers containing ELC fluorescently labeled at different sites in their C-terminal half, which showed a significant decrease in fluorescence lifetime of ELC probes upon stretch of fibers [13].

Thus, the previous works suggested that the rotation of the regulatory domain of the myosin head relative to the motor domain occurring during the ATPase cycle can be accompanied by a rather tight interaction between the motor domain and ELC associated with the regulatory domain. However the details of this interaction were not investigated. The main goal of the present research was to obtain additional experimental evidence on this interaction. For this purpose, we applied differential scanning calorimetry (DSC) combined with temperature dependences of fluorescence to study thermal unfolding and the domain structure of S1 in the ternary complexes S1-ADP-AlF_4_^-^ and S1-ADP-BeF_x_ mimicking the S1 ATPase intermediate states S1**-ADP-P_i_ and S1*-ATP.

DSC is the most effective and commonly employed method to study thermal unfolding of proteins [14], which represents a powerful experimental approach for probing global nucleotide-induced structural changes in the myosin head. It has previously been shown that the formation of the ternary complexes S1-ADP-V_i_, S1-ADP-BeF_x_, and S1-ADP-AlF_4_ (i.e. stable analogs of the intermediate states of the S1-catalyzed Mg^2+^-ATPase reaction [15–17]) causes a global change of S1 conformation, which is expressed in a pronounced increase of S1 thermal stability and in a significant change of S1 domain structure [18–24]. Besides that, the DSC method is one of the best approaches for revealing structural domains in multidomain proteins as distinct thermal transitions on the heat sorption curve corresponding to the regions in a protein molecule which unfold cooperatively and independently from each other [14, 25]. To disclose such domains in the myosin head, in early works, made more than 20 years ago, the so-called “successive annealing” method was applied in DSC studies on the thermal unfolding of skeletal S1, which revealed three separate transitions (calorimetric domains) in the S1 molecule [18, 22, 26, 27]. More recently, a new calorimetric approach was specifically developed to analyze the DSC profiles of irreversibly denaturing multidomain proteins, and this approach was applied for detailed analysis of the domain structure of nucleotide-free S1 [28]. Using this approach, we revealed two calorimetric domains in the S1 molecule, where the more thermostable domain denatured in two steps. This calorimetric domain was identified as the S1 motor domain by comparing the DSC data with the temperature dependences of intrinsic fluorescence parameters and S1 ATPase inactivation (note that all Trp residues as well as the ATPase site are located within the S1 motor domain) [28]. In the present work, we applied this approach to investigate the changes occurring in the S1 domain structure upon formation of the ternary complexes S1-ADP-AlF_4_^-^ and S1-ADP-BeF_x_. To identify on the DSC profile thermal transitions corresponding to the S1 regulatory domain, we analyzed temperature-induced changes in fluorescence of recombinant ELC mutants (light chain 1 isoform), which were first fluorescently labeled at different positions in their C-terminal half and then introduced into the S1. The results testify in favor of the proposed nucleotide-induced interaction between the motor domain and ELC, which leads to a significant increase in thermal stability of the S1 regulatory domain.

## Materials and Methods

### Materials

The most standard chemicals and 5-(iodoacetamidoethyl)amino-naphtalene-1-sulfonic acid (1,5-IAEDANS) were purchased from Sigma. All chemicals were of the highest purity and quality available. All solutions in the study were prepared on the milliQ-quality water (18.3 MΩ/cm) and filtered through the 0.22 μm Millipore filter system before use.

### Proteins

The previously described DNA construct of human ventricular LC1 containing the His tag at the C-terminus and a single cysteine residue (Cys-180) near the C-terminus [13] was used to generate several mutant constructs containing single cysteine residues at different positions in the C-terminal half of the protein. First, a cysteine-free sequence was produced in which the wild-type Cys-180 was changed to alanine. Using this sequence, four constructs were obtained by mutating Ser-99, Thr-127, Gly-142, and Glu-160 to cysteines as described earlier [13]. The LC1 was then overexpressed in M15(pREP4) *Escherichia coli* cells and purified using affinity chromatography on a HisTrap HP 5 ml column (Amersham Pharmacia). Native LC1 (without a His-tag) was prepared from rabbit skeletal muscle myosin as described by Holt and Lowey [29]. The concentration of LC1 was determined spectrophotometrically using extinction coefficient *A* ^1%^ at 280 nm of 2.0 cm^−1^.

S1 from rabbit skeletal myosin was prepared by digestion of myosin filaments with TLCK-treated α-chymotrypsin (Sigma) as described earlier [30]. The concentration of S1 was estimated spectrophotometrically using extinction coefficient *A* ^1%^ at 280 nm of 7.5 cm^−1^. Small heat shock protein HspB5 (αB-crystallin) preparation was a gift from Prof. Nikolai B. Gusev (Department of Biochemistry, School of Biology, Moscow State University, Moscow, Russia).

### Protein labeling

Each isolated LC1 was labeled by incubation with a 5-fold molar excess of 1,5-IAEDANS for 4 h at 4°C in a 10 mM Na-phosphate buffer (pH 7.0) containing 100 mM NaCl. The unreacted dye was removed by exhaustive dialysis against 50 mM imidazole-HCl buffer (pH 7.0) containing 100 mM NaCl and 5 mM DTT. The labeling efficiency was assessed spectrophotometrically using the extinction coefficients of 1,5-IAEDANS equal to 5.7^.^10^3^ mol^-1^ cm^-1^ at 337 nm and 1.03^.^10^3^ mol^-1.^cm^-1^ at 280 nm. Typically, more than 85% of the LC1 was labeled.

The reactive SH-group of Cys-707 in the S1 heavy chain (referred to as SH1) was labeled by incubating S1 with 1,5-IAEDANS at 4°C for 24 h, at a molar ratio of S1 to dye equal to 1:1. The extent and specificity of SH1 labeling was controlled by inhibition of the NH_4_^+^-EDTA-ATPase and activation of the Ca^2+^-ATPase in the presence of 0.6 M KCl [21, 31]. In our experiments, more than 95% of the SH1 groups were specifically modified.

### Exchange of LC1 in S1

Fluorescently labeled LC1 was exchanged into S1 as described by Zaager and Burke [32]. The exchange was performed by incubation of S1 with a 8-fold molar excess of free LC1 in 50 mM imidazole-HCl buffer (pH 7.0) containing 5 mM DTT, 10 mM MgATP and 100 mM NaCl at 37°C for 30 min. The reaction was stopped by cooling on ice. S1 was purified on a SP-trisacryl column to separate exchanged S1(LC1) from free LC1 and unexchanged S1 [33]. The concentration of labeled S1 was calculated from the extinction coefficients of S1 at 280 nm and 1,5-IAEDANS at 337 nm and 280 nm. Typically, 30–40% of the S1 was labeled.

### Preparation of stable ternary complexes S1-ADP-BeF_x_ and S1-ADP-AlF_4_

Trapping of ADP by different phosphate analogs (BeF_x_ or AlF_4_) was performed as described earlier [16, 17, 19–22, 31]. To form stable ternary complexes S1-ADP-BeF_x_ and S1-ADP-AlF_4_, S1 was incubated with 0.7 mM MgADP and 5 mM NaF for 10 min at 20°C prior to addition of BeSO_4_ or AlCl_3_, respectively, to a final concentration of 0.5 mM. Then the solution was incubated overnight on ice. The formation of the complexes was controlled by measuring the NH_4_^+^-EDTA-ATPase activity of S1. The ATPase activity of S1 in the S1-ADP-BeF_x_ and S1-ADP-AlF_4_ complexes did not exceed 5–7% of the activity measured in the absence of P_i_ analogs.

### Differential Scanning Calorimetry (DSC)

DSC experiments were performed on a DASM-4M differential scanning microcalorimeter (Institute for Biological Instrumentation, Pushchino, Russia) as described earlier [18–23, 28, 31]. The S1 samples (1 mg/mL) were heated with a constant rate of 1 K/min from 15°C to 80°C in a 30 mM Hepes-KOH (pH 7.3) buffer containing 2 mM MgCl_2_ and 100 mM NaCl. The protein samples were reheated after the first scan and subsequent cooling to check the reversibility of thermal denaturation. The denaturation of all S1 species was fully irreversible. All calorimetric traces were corrected for instrumental background and subjected to time response correction as described in [28] before further analysis. Decomposition of S1 thermograms into individual thermal transitions was performed using specifically developed calorimetric approach intended for analysis of the DSC profiles of irreversibly denaturing multidomain proteins, that was described in detail in [28]. Briefly, this approach was based on annealing procedures which were performed by careful pre-heating the protein samples at an appropriate temperature for a definite period of time immediately before DSC measurements (the temperature and time for these procedures were calculated as described in the previous work [28]).

According to this approach, each calorimetric transition is assumed to be a pseudo-first- order irreversible reaction characterized by two parameters: calorimetric enthalpy (Δ*H*_cal_) and kinetic rate constant (*k*(*T*)). The first of them (Δ*H*_cal_) is assumed to be temperature-independent, and the second (*k*(*T*)) depends on temperature according to Arrhenius law:

$$k=\text{exp}\left\{\frac{E_{a}}{R}(\frac{1}{T*}-\frac{1}{T})\right\}$$

where *E*_a_ is the experimental activation energy (temperature-independent), *R* is the universal gas constant, *T* is the absolute temperature, and T* is the temperature at which *k* = 1 min^−1^ (the dimension of *k*(*T*) is min^−1^). Thus, three temperature-independent parameters, namely Δ*H*_cal_, *E*_a_ and T*, completely describe each calorimetric transition. As to calorimetric domains, each of them can include one or more calorimetric transitions. In the latter case, these transitions are thought to be successive, i.e. thermal denaturation of the domain includes kinetic intermediates [28].

### Fluorescence measurements

Fluorescence studies were performed on a Cary Eclipse spectrofluorimeter (Varian) equipped with a Peltier-controlled cell holder and thermoprobes. All measurements were performed in a 30 mM Hepes-KOH buffer (pH 7.3) containing 2 mM MgCl_2_ and 100 mM NaCl at a protein concentration of 0.15–0.2 mg/mL. Intrinsic tryptophan fluorescence of S1 was excited at 297 nm (slit width 5 nm) and recorded in the range of 310–450 nm (slit width 2.5 nm). Fluorescence of AEDANS attached to S1 was excited at 365 nm (slit width 5 nm) and recorded in the range of 385–600 nm (slit width 2.5 nm). The protein samples were heated with a constant rate of 1°C/min (i.e. at the same heating rate as for DSC experiments) from 20 to 70°C, and the fluorescence intensities at 320 nm and 365 nm were recorded for intrinsic tryptophan fluorescence and at 450 nm and 535 nm—for fluorescence of AEDANS. The position and form of the tryptophan fluorescence spectra were characterized by parameter *A* (*A* = *I*_320_/*I*_365_ where *I*_320_ and *I*_365_ are fluorescence intensities at λ_em_ = 320 and 365 nm, respectively) [34, 35]. Upon heating, the spectrum of S1 tryptophan fluorescence was shown to shift towards shorter wavelengths, and this spectral shift was accompanied by a pronounced increase in values of the parameter *A* [28]. This indicated that the environment of tryptophan residues (at least some of them) became more hydrophobic during irreversible thermal denaturation of S1. Here we applied similar approach to characterize the thermally induced changes in the extrinsic fluorescence of the label specifically bound to LC1 in the S1 molecule. For this purpose we measured the temperature-induced changes in a so-called “parameter *L*” (from “Label”), which is the ratio *I*_450_/*I*_535_, where *I*_450_ and *I*_535_ are fluorescence intensities of AEDANS at λ_em_ = 450 and 535 nm, respectively. The temperature dependences of the parameters *A* and *L* were analyzed by using OriginPro 9.0 software (OriginLab Corp., Northampton, MA) as follows. Experimental data were normalized and fitted with the simplest sigmoidal function (so-called “fit Boltzmann” in Origin's internal documentation) that describes transition of normalized parameter *A* or *L* (*P*_norm_) from 0 to 1 as temperature (*T*) grows: *P*_norm_(*T*) = 1–1/[1 + exp((*T*–*T*_0.5_)/*d*)]. Two parameters obtained from this fitting were *T*_0.5_, the transition midpoint (i.e. the temperature at which a 50% increase in the normalized parameters *A* and *L* occurs), and *d* which reflects the curve slope at a transition midpoint and the width of the temperature range in which the changes in the fluorescence parameters occur.

### Heat-induced dissociation of ELC from the S1 heavy chain

This approach was based on the fact that irreversible thermal denaturation of S1 is accompanied by its aggregation which can be detected by a significant increase in either turbidity or light scattering [18, 28, 36]. S1 (0.5 mg/ml) in the absence of nucleotides or in the S1-ADP-BeF_x_ and S1-ADP-AlF_4_ complexes was heated with a constant rate of 1°C/min in the medium containing 30 mM Hepes (pH 7.3), 2 mM MgCl_2_, and 100 mM NaCl., and the S1 aggregation was recorded by an increase in light scattering intensity at 350 nm on a Cary Eclipse spectrofluorimeter (Varian). During the heating, 100 μL aliquots of the sample were taken, cooled down, and subjected to ultracentrifugation at 140,000 g for 20 min on a Beckman airfuge (Beckman Instruments Inc., USA). The protein composition of supernatants obtained was analyzed by SDS–PAGE [37]. Quantification of protein bands was carried out by densitometry using an Astra 6700 scanner (UMAX) and scanned images were analyzed using the ImageJ 1.45s software (Scion Corp., Frederick, MD).

## Results

### Thermal denaturation of myosin S1 in the ternary complexes S1-ADP-BeF_x_ and S1-ADP-AlF_4_

In the previous work we applied DSC for a detailed comparative analysis of thermal denaturation and domain structure of two S1 isoforms containing different ELC (LC1 and LC3, also termed as “alkali” light chains A1 and A2) in the nucleotide-free state. We revealed two calorimetric domains in the S1 molecule, where the more thermostable domain in turn denatured in two steps. Some difference between the two S1 isoforms was only revealed by DSC in thermal denaturation of the least thermostable domain, within the temperature range of 35–45°C [28], suggesting that this calorimetric domain may correspond to the S1 regulatory domain containing ELC. In further studies we also applied special approaches to identify these calorimetric domains, *i*.*e*., to define their correspondence to structural domains of the S1 molecule. For this purpose the temperature dependences of intrinsic fluorescence parameters and S1 ATPase inactivation were recorded at the same heating rate (1°C/min) as for DSC experiments and were compared with the DSC data (note that all Trp residues as well as the ATPase site are located within the S1 motor domain). Using this approach, we have identified the more thermostable calorimetric domain as the S1 motor domain, because changes in fluorescence and ATPase activity were only observed within the same temperature range as this domain on the DSC profile. On the other hand, thermal denaturation of the least thermostable domain was not accompanied by any changes in fluorescence and activity, and therefore we proposed that this calorimetric domain (which melts at ~40°C) can be assigned to the S1 regulatory domain which does not contain tryptophan residues [28]. Here we applied the same approach to explore the domain structure of S1 in the ternary complexes S1-ADP-AlF_4_^-^ and S1-ADP-BeF_x_ (with the exception of ATPase measurements as S1 has no ATPase activity in these complexes).

**Fig 2:**
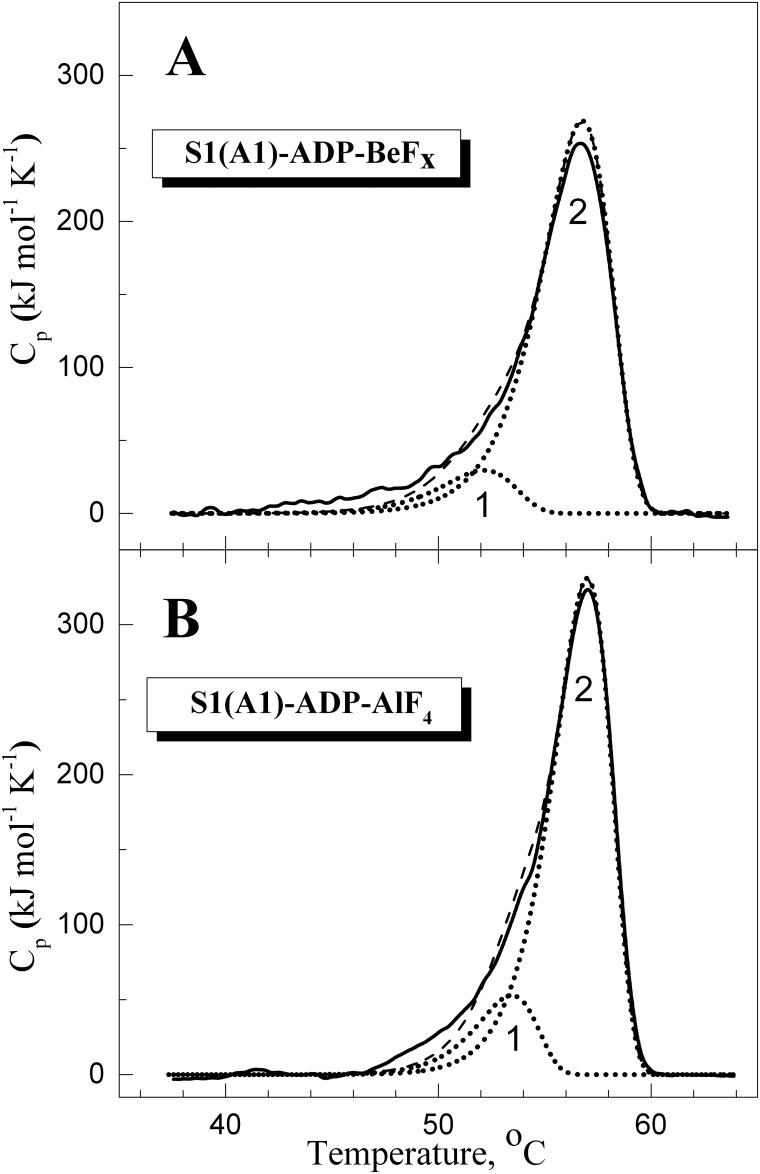
Temperature dependence of the excess molar heat capacity (C_p_) obtained by DSC for S1 in the complexes S1-ADP-BeF_x_ (A) and S1-ADP-AlF_4_^-^ (B), and their decomposition into individual thermal transitions (calorimetric domains 1 and 2). Decomposition of the DSC curves (solid lines) into individual thermal transitions (dotted lines) was performed as described earlier for the nucleotide-free S1 [28]. The curves shown by dashed lines represent the results of the fitting of the DSC data in a framework of the two-domain model [28]. The values of calorimetric enthalpy (Δ*H*_cal_) for each thermal transition are presented in Table 1.

**Fig 3:**
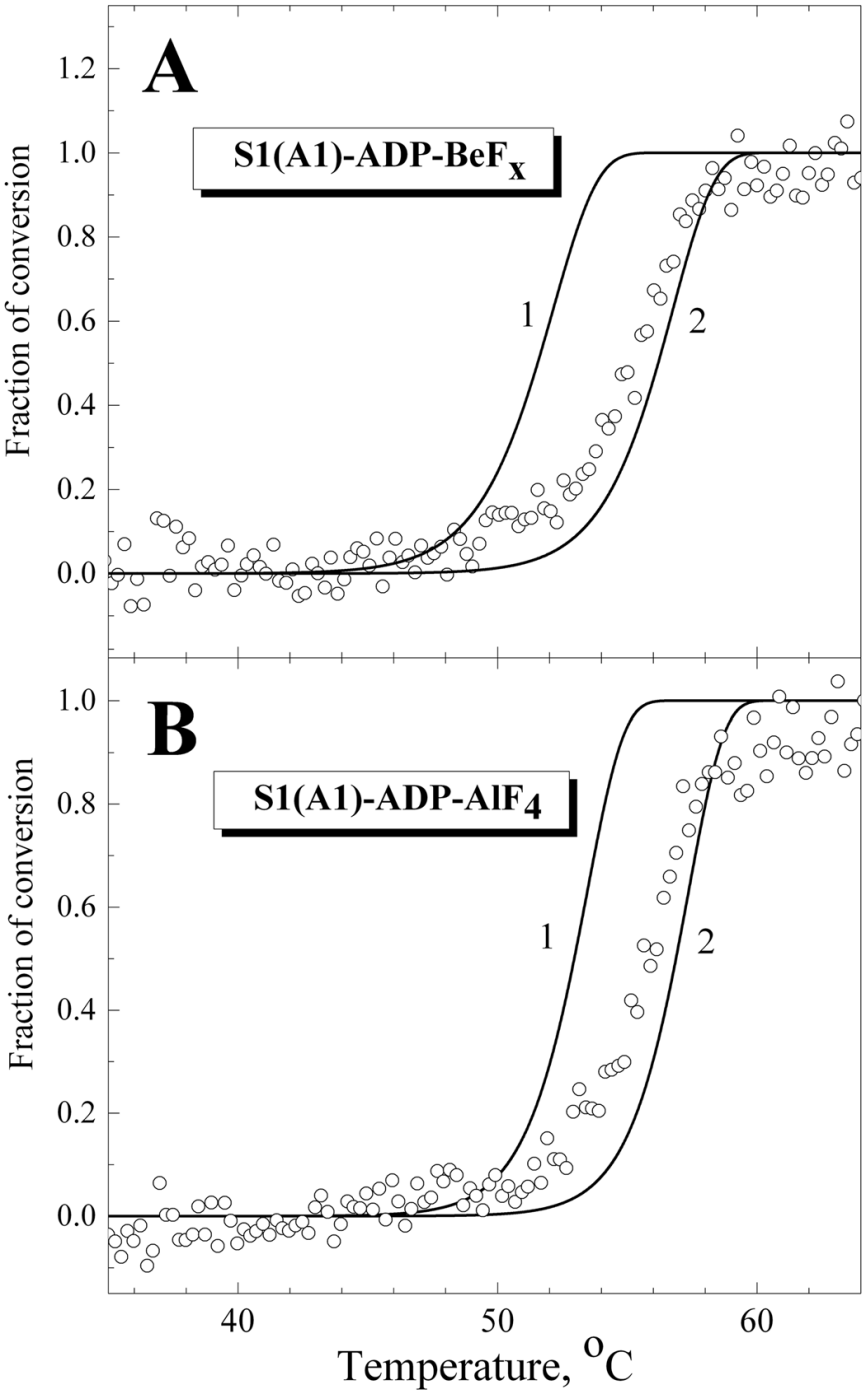
Temperature-induced changes of the intrinsic tryptophan fluorescence for S1 in the complexes S1-ADP-BeF_x_ (A) and S1-ADP-AlF_4_^-^ (B) in comparison with fraction of conversion for the calorimetric transitions 1 and 2. The changes in the S1 intrinsic fluorescence were measured by the changes of the value of normalized parameter *A* (open circles), which was calculated as described in Materials and methods. The fraction of conversion for each calorimetric transition (solid lines) was simulated using mean values of parameters *E*_a_ and T* presented in Table 1.

Fig 2 shows the DSC curves obtained for S1 in the complexes S1-ADP-BeF_x_ (Fig 2A) and S1-ADP-AlF_4_^-^ (Fig 2B), and their decomposition into individual thermal transitions (calorimetric domains). It is seen that in both cases the DSC curve can be decomposed into two such transitions. Each of these thermal transitions obtained from DSC experiments is presented in Fig 3 as a fraction of conversion from the native to the denatured state, and their temperature dependences are compared with those of normalized parameter *A* which reflects the temperature-induced changes of the S1 tryptophan fluorescence. It is clearly seen that, for both complexes, S1-ADP-BeF_x_ and S1-ADP-AlF_4_^-^ (Fig 3A and 3B), the temperature-induced changes of the parameter *A* almost coincide with the curve corresponding to the calorimetric domain 2, whereas no changes of the parameter *A* are observed in the range of thermal denaturation of the calorimetric domain 1. Taking into account the fact that all tryptophan residues are localized in the motor domain of the S1 molecule, our results allow to identify the calorimetric domain 2 as the S1 motor domain, while the calorimetric domain 1 can be assigned to the S1 regulatory domain which does not contain tryptophan residues. The main calorimetric parameters extracted from these data for each thermal transition (calorimetric enthalpy, Δ*H*_cal_, activation energy, *E*_a_, T*, and the transition midpoint, *T*_0.5_), as well as *T*_0.5_ values for the parameter *A*, are summarized in Table 1 and compared with those obtained earlier for the nucleotide-free S1 [28]. (Note that for nucleotide-free S1 transitions 2 and 3 represent two successive stages of denaturation of calorimetric domain 2, and transition 1 corresponds to calorimetric domain 1 [28], whereas for the S1-ADP-BeF_x_ and S1-ADP-AlF_4_^-^ complexes each thermal transition corresponds to separate calorimetric domain.).

**Table 1:** The main parameters extracted from the DSC data for each thermal transition of S1 in the absence of added nucleotides and in the complexes S1-ADP-BeF_x_ or S1-ADP-AlF_4_^-^ (Δ*H*_cal_, *E*_a_, T*, and *T*_0.5_), as well as from temperature-induced changes of normalized parameter *A* (*T*_0.5_).

Parameter	DSC				Fluorescence
	Δ*H*_cal_ (kJ/mol)^a^	*E*_a_ (kJ/mol)^a^	T* (K)^a^	*T*_0.5_ (°C) ^b^	*T*_0.5_ for parameter *A*(°C) ^b^
**Nucleotide-free S1** ^c^					
transition 1	200	290	317.5	40.3	
transition 2	1030	400	321.2	45.7	47.0
transition 3	500	340	324.5	49.2	
**S1-ADP-BeF**_**x**_					
transition 1	140	515	326.2	51.5	
transition 2	1170	560	330.7	56.2	55.5
**S1-ADP-AlF**_**4**_					
transition 1	200	630	327.1	53.0	
transition 2	1210	670	330.6	56.5	55.8

^a^ The values of Δ*H*_cal_, *E*_a_, and T* were calculated as described in [28]. The relative error of the given values of calorimetric enthalpy, Δ*H*_cal_, did not exceed ± 10%. The error of the calculated values of activation energy, *E*_a_, and T* was equal to ±40 kJ/mol and ±0.4 K, respectively.

^b^ The values of *T*_0.5_ for each calorimetric transition and for parameter *A* were extracted from Fig 3. The absolute error of the given *T*_0.5_ values did not exceed ± 0.5°C.

^c^ The data for nucleotide-free S1 were taken from [28].

It has previously been shown that the formation of the ternary complexes S1-ADP-BeF_x_ and S1-ADP-AlF_4_ causes a global change of S1 conformation, which is expressed in a pronounced increase of S1 thermal stability [22, 24, 31]. Comparing the data presented in Table 1 for these complexes with those obtained earlier for the nucleotide-free S1 [28], one can conclude that formation of these complexes drastically increases the thermal stability of transition 1 by shifting this transition by 10–12°C towards higher temperature. Taking into account the fact that this transition is not accompanied by changes in tryptophan fluorescence (Fig 3), we can assume that it corresponds to the thermal unfolding of the S1 regulatory domain which does not contain tryptophan residues. If so, the above results may indicate that the formation of the ternary complexes S1-ADP-BeF_x_ and S1-ADP-AlF_4_ is accompanied by a rather tight interaction between the two main domains of the S1 molecule, the regulatory domain and the motor domain, and this interaction leads to a significant increase in the thermal stability of the regulatory domain. In favor of this assumption is also a significant increase in the activation energy, *E*_a_, for transition 1 in the S1-ADP-BeF_x_ and S1-ADP-AlF_4_ complexes in comparison with nucleotide-free S1 (Table 1), which may reflect an increase in the number of bonds stabilizing the regulatory domain.

However, it should be noted that this assumption is based on the indirect data, as the assignment of the least thermostable transition 1 to the S1 regulatory domain is deduced only from the fact that this domain does not contain tryptophan residues. To be sure that the thermal transition 1, whose stability strongly increases upon formation of the complexes S1-ADP-BeF_x_ and S1-ADP-AlF_4_, indeed corresponds to the thermal unfolding of the S1 regulatory domain, it would be much better to have direct data, i.e. to obtain information on the unfolding of the regulatory domain directly from this domain.

For this purpose, we used several recombinant ELC (LC1 isoform) mutants which were fluorescently labeled at a single cysteine residue at different positions in the C-terminal half of the LC1 molecule and then exchanged into S1. The temperature induced changes in the fluorescence parameters of these LC1 mutants associated with the S1 regulatory domain, which provided information on the thermal unfolding of this domain, were then compared with the corresponding DSC data and with the changes in tryptophan fluorescence, which reflected thermal unfolding of the S1 motor domain.

### Temperature dependences of fluorescence of labeled LC1 associated with the S1 regulatory domain

#### Comparison of recombinant LC1 with native LC1

**Fig 4:**
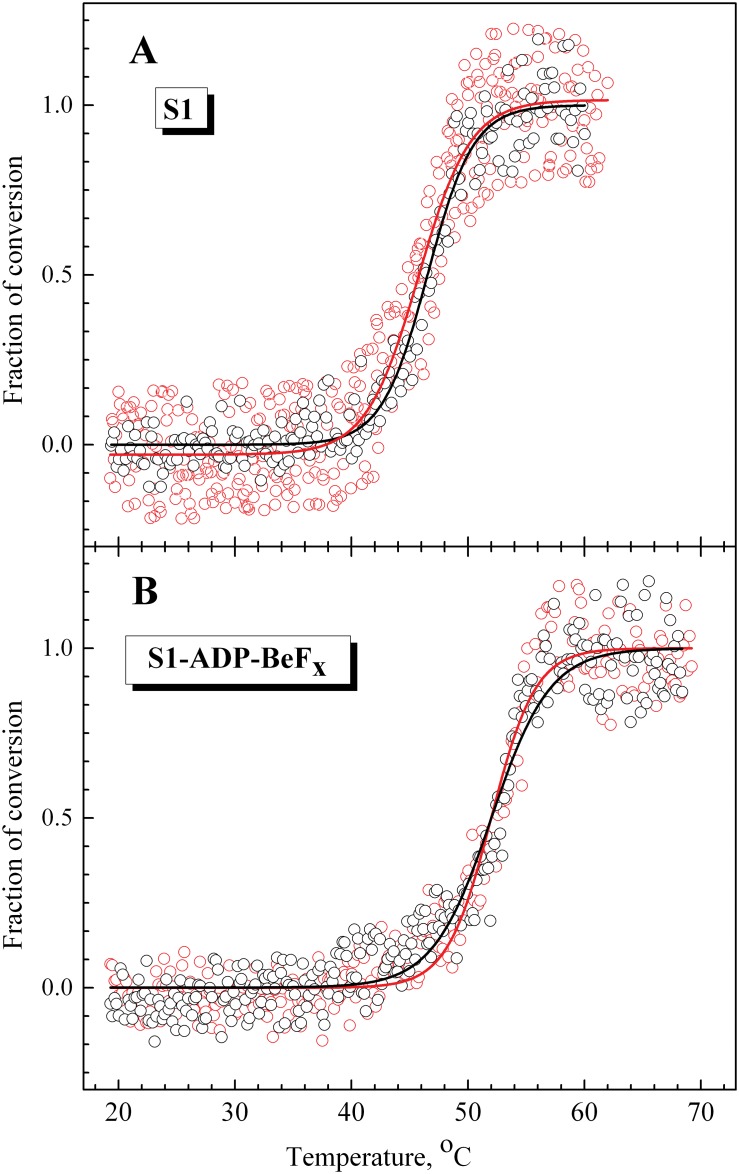
Temperature-induced changes in fluorescence of labeled native (black circles) and recombinant (red circles) LC1 associated with the S1 regulatory domain in nucleotide-free S1 (A) or in the complex S1-ADP-BeF_x_ (B). The changes in the LC1 fluorescence were measured by the changes of the value of normalized parameter *L*, which was calculated as described in Materials and methods. Black and red solid lines represent the results of the data analysis according to a sigmoidal function (Boltzmann) for the native and recombinant wild-type LC1, respectively.

Each recombinant LC1 contained the His-tag at the C-terminus. It cannot be excluded that the presence of the His-tag may affect the properties of fluorescently labeled LC1 associated with the S1 regulatory domain. To check this, first of all we compared the temperature dependences of fluorescence of recombinant wild-type LC1 bound to the S1 heavy chain with those for native, tag-free LC1 prepared from rabbit skeletal muscle myosin. Both these LC1 species contained a single cysteine residue (Cys-180) near the C-terminus and were labeled by AEDANS under similar conditions and then exchanged into S1. The temperature dependences of parameter *L* for fluorescence of labeled LC1 in S1 were almost identical for both these LC1 species, both for nucleotide-free S1 (*T*_0.5_ = 46 ± 0.5°C) (Fig 4A) and for S1 in the S1-ADP-BeF_x_ complex (*T*_0.5_ = 52 ± 0.5°C) (Fig 4B). Thus, we can conclude that the presence of the His-tag at the C-terminus of LC1 has little to no influence on the thermally-induced changes in fluorescence of labeled LC1 associated with the S1 regulatory domain and therefore will not interfere with the following experiments.

#### Thermally induced changes in fluorescence of labeled LC1 mutants associated with the regulatory domain of the nucleotide-free S1

**Fig 5:**
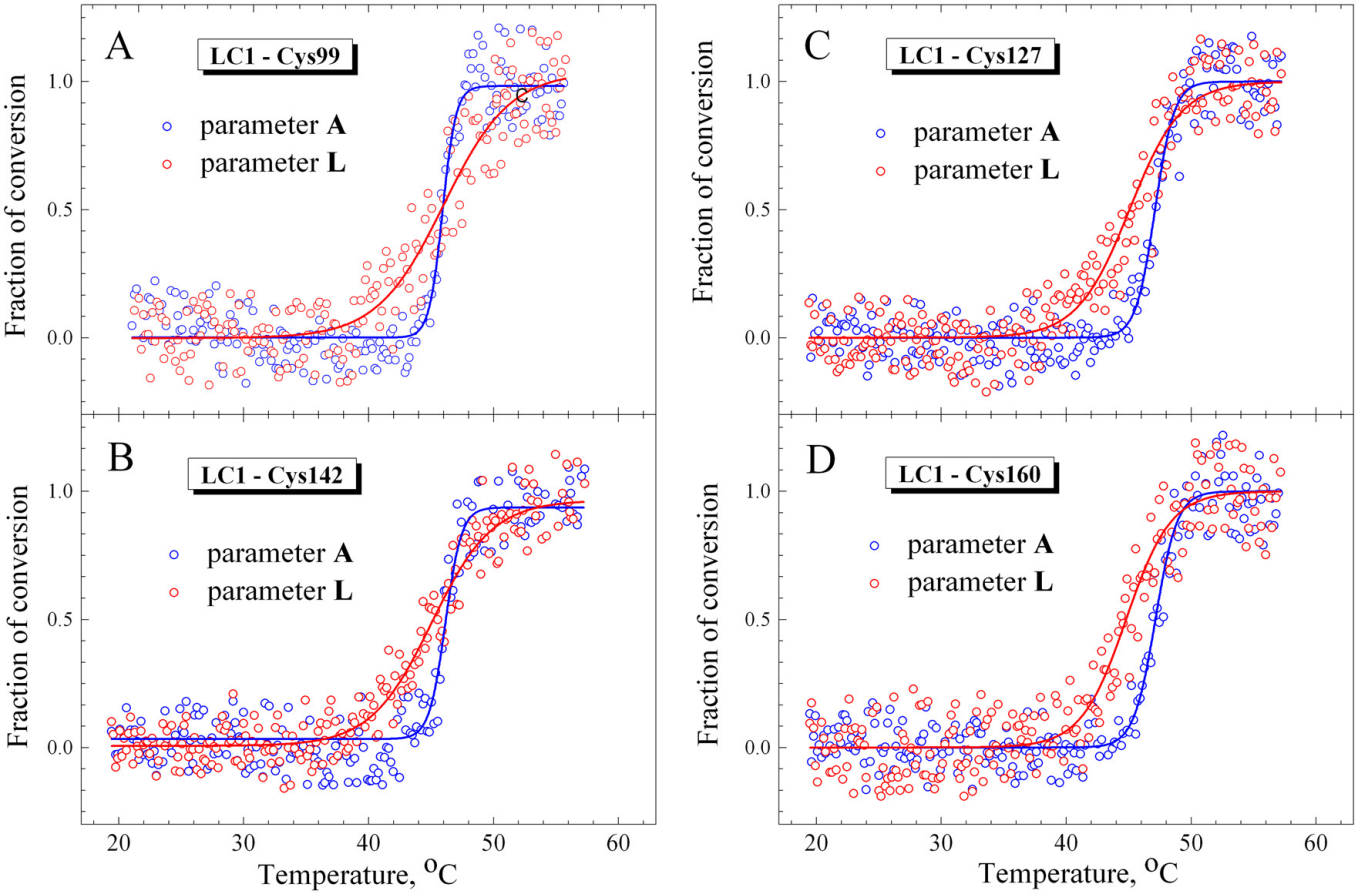
Temperature-induced changes in fluorescence measured on the nucleotide-free S1 for labeled recombinant LC1 mutants associated with the S1 regulatory domain (red circles) and for the tryptophan fluorescence of S1 (blue circles). The labeled Cys residues in the LC1 were Cys-99 (A), Cys-142 (B), Cys-127 (C), and Cys-160 (D). The changes in fluorescence were measured by the changes of values of normalized parameters *L* and *A* for the AEDANS-labeled LC1 and tryptophan residues, respectively, calculated as described in Materials and methods. Red and blue solid lines represent the results of the data analysis according to a sigmoidal function (Boltzmann) for LC1 and tryptophan, respectively.

We prepared several recombinant LC1 mutants, each containing a single cysteine residue at different positions in the C-terminal half of the LC1 molecule (Cys-99, Cys-127, Cys-142, Cys-160, Cys-180), which were fluorescently labeled at these cysteines with 1,5-IAEDANS and then exchanged into S1. Prior to the experiments on the S1-ADP-BeF_x_ and S1-ADP-AlF_4_ complexes, we studied the temperature dependences of fluorescence of these LC1 mutants both free in solution and bound to the regulatory domain of the nucleotide-free S1. Isolated LC1 species did not demonstrate any changes in the fluorescent parameter *L* upon heating (data not shown). Temperature-induced changes in parameter *L* for fluorescence of these LC1 mutants associated with the regulatory domain of nucleotide-free S1 are presented on Fig 5 in comparison with the changes in parameter *A* for the tryptophan fluorescence, which reflect thermal unfolding of the S1 motor domain. The values of *T*_0.5_ and *d* extracted from the analysis of the temperature-induced changes in parameters *A* and *L* are summarized in Table 2.

**Table 2:** The main parameters, *T*_0.5_ and *d*, obtained from the analysis of the temperature-induced changes in the fluorescent parameters *A* and *L* for tryptophan and AEDANS-labeled LC1 mutants, respectively. The data were obtained from experiments on the nucleotide-free S1 (Fig 5).

Cys position in LC1	parameter *A*		parameter *L*	
	*T*_0.5_ (°C)	*d*	*T*_0.5_ (°C)	*d*
**Cys-180**	47.0	0.94	45.7	2.1
**Cys-160**	47.0	0.88	44.7	1.85
**Cys-142**	46.2	0.68	45.2	2.4
**Cys-127**	47.0	0.88	45.0	2.29
**Cys-99**	46.5	0.59	46.4	2.6

The absolute error of the given *T*_0.5_ values did not exceed ± 0.5°C for parameter *A* and ± 1.0°C for parameter *L*. The absolute error of the given *d* values did not exceed ± 0.1 for parameter *A* and ± 0.25 for parameter *L*.

The results presented in Fig 5 and Table 2 show that for most LC1 mutants the changes in parameter *L* occur at somewhat lower temperature than the changes in parameter *A*. In most cases, with the only exception of LC1 Cys-99 mutant, the *T*_0.5_ value for parameter *L* is lower, by 1–2°C, than that for parameter *A* (Table 2). It should also be noted that in all cases the slope of the curve for the parameter *L* is more gentle than for the parameter *A* (Fig 5); as a result, the *d* value for the parameter *L* significantly exceeds this value for parameter *A* (Table 2). This difference in the *d* values is mainly the result of the difference between the temperatures at which the fluorescent parameters begin to change: the parameter *L* starts to rise at lower temperature (~37–40°C) than the parameter *A* (~45°C) (Fig 5). At the same time, no appreciable changes in the parameter *L* were observed at 40°C, i.e. the temperature at which the thermal denaturation of the regulatory domain in the nucleotide-free S1 was predicted [28]. These results imply that the thermal unfolding of the S1 regulatory domain containing associated LC1 is a rather complicated process. For example, the unusual behavior of parameter *L* (Fig 5) might be caused by protein aggregation which usually accompanies thermal denaturation of S1 [28, 36, 38]. To check such a possibility, we have tried to separate the process of the S1 thermal denaturation from its aggregation by using small heat shock protein HspB5 (αB-crystallin).

#### Temperature induced changes in fluorescence and light scattering of S1 in the presence or absence of the small heat shock protein HspB5

**Fig 6:**
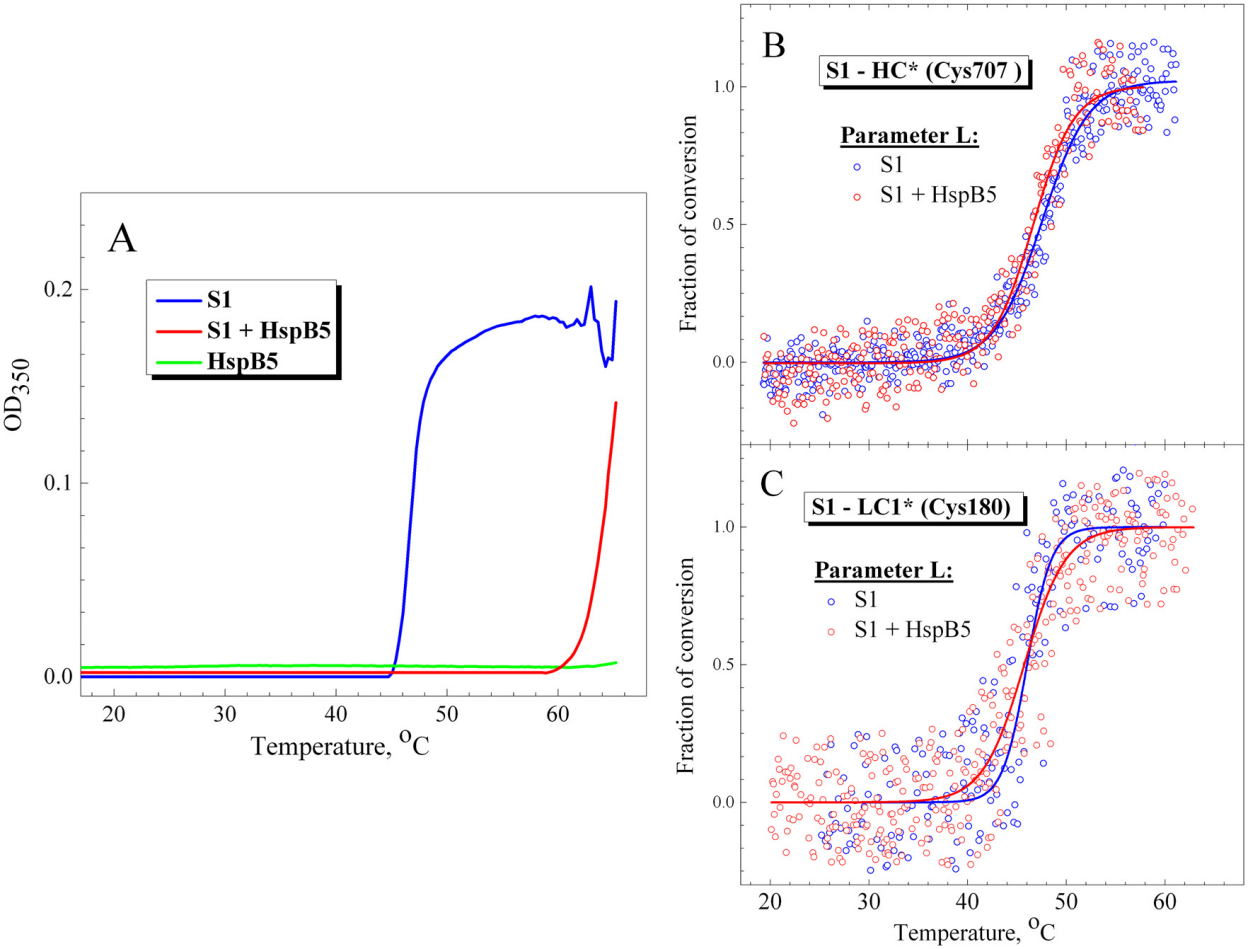
Temperature dependences obtained in the presence or absence of HspB5 for S1 aggregation (A) and for fluorescence of labels specifically attached to Cys-707 in the S1 motor domain (B) or to Cys-180 of the wild-type LC1 in the S1 regulatory domain (C). (**A**) S1 aggregation was measured by an increase in turbidity (apparent optical density at 350 nm) of the solution in the absence (blue curve) or in the presence (red curve) of HspB5. Green curve represents the optical density of HspB5 in the absence of S1. (**B,C**) The changes in the fluorescence were measured by changes of the values of normalized parameter *L* for fluorescent label (IAEDANS) attached to the motor (B) or regulatory (C) domain of the S1 molecule. The changes of parameter *L* in the absence or in the presence of HspB5 are shown by blue and red circles, respectively. Correspondingly, blue and red solid lines represent the results of the data analysis according to a sigmoidal function (Boltzmann). Protein concentration was 0.15 mg/mL for both S1 and HspB5. Heating rate was 1°C/min.

Previous studies have shown that the small heat shock protein HspB1 (Hsp27) effectively prevents the heat-induced aggregation of S1 with almost no effect on the S1 thermal unfolding measured by DSC [36]. Here we used another small heat shock protein, HspB5 (αB-crystallin), to suppress the S1 aggregation induced by its thermal denaturation. We studied the temperature dependences of S1 aggregation by measuring an increase in the apparent optical density or light scattering of the protein, and compared them with the temperature dependences of fluorescent parameter *L* registered either in the absence or in the presence of HspB5. The results showed that HspB5 effectively prevents the thermally-induced aggregation of S1 by shifting the aggregation curve by ~20°C towards higher temperature (Fig 6A). As a result, S1 begins to aggregate in the presence of HspB5 at much higher temperature (above 60°C), i.e. under conditions when the S1 thermal denaturation is already completed. In contrast, we did not observe any appreciable influence of HspB5 on the temperature dependences of fluorescent parameter *L* (Fig 6B and 6C). In this case, we could not analyze the tryptophan fluorescence of S1 as HspB5 on its own contains two tryptophan residues [39]. Therefore, we analyzed the temperature dependences of parameter *L* for the fluorescent label (IAEDANS) specifically attached to the reactive SH1 group (Cys-707 in the S1 heavy chain) located in the S1 motor domain. These dependences obtained in the absence or presence of HspB5 (Fig 6B) were identical (*T*_0.5_ was equal to 47 ± 0.5°C for both) and very similar to those obtained for parameter *A* of the S1 tryptophan fluorescence (Fig 5, Table 2). The temperature dependences of parameter *L* for the fluorescent label attached to Cys-180 of the wild-type LC1 in the S1 regulatory domain were also almost identical regardless of the presence of HspB5 (Fig 6C). Thus, effectively preventing the heat-induced S1 aggregation (Fig 6A) HspB5 has practically no effect on the temperature-induced changes in the S1 fluorescence (Fig 6B and 6C), which reflect the thermal unfolding of the motor and regulatory domains of the S1 molecule.

These results may indicate that S1 aggregation usually accompanying its thermal denaturation has no appreciable effect on the temperature-induced structural changes in the motor and regulatory domains of the S1 molecule. Therefore, the temperature-induced changes in the fluorescent parameters, which are observed in our experiments, most likely reflect the thermal denaturation of the S1 domains, but not the consequent aggregation of the protein.

#### Thermally induced changes in fluorescence of labeled LC1 mutants associated with the regulatory domain of S1 in the ternary complexes S1-ADP-BeF_x_ and S1-ADP-AlF_4_

**Fig 7:**
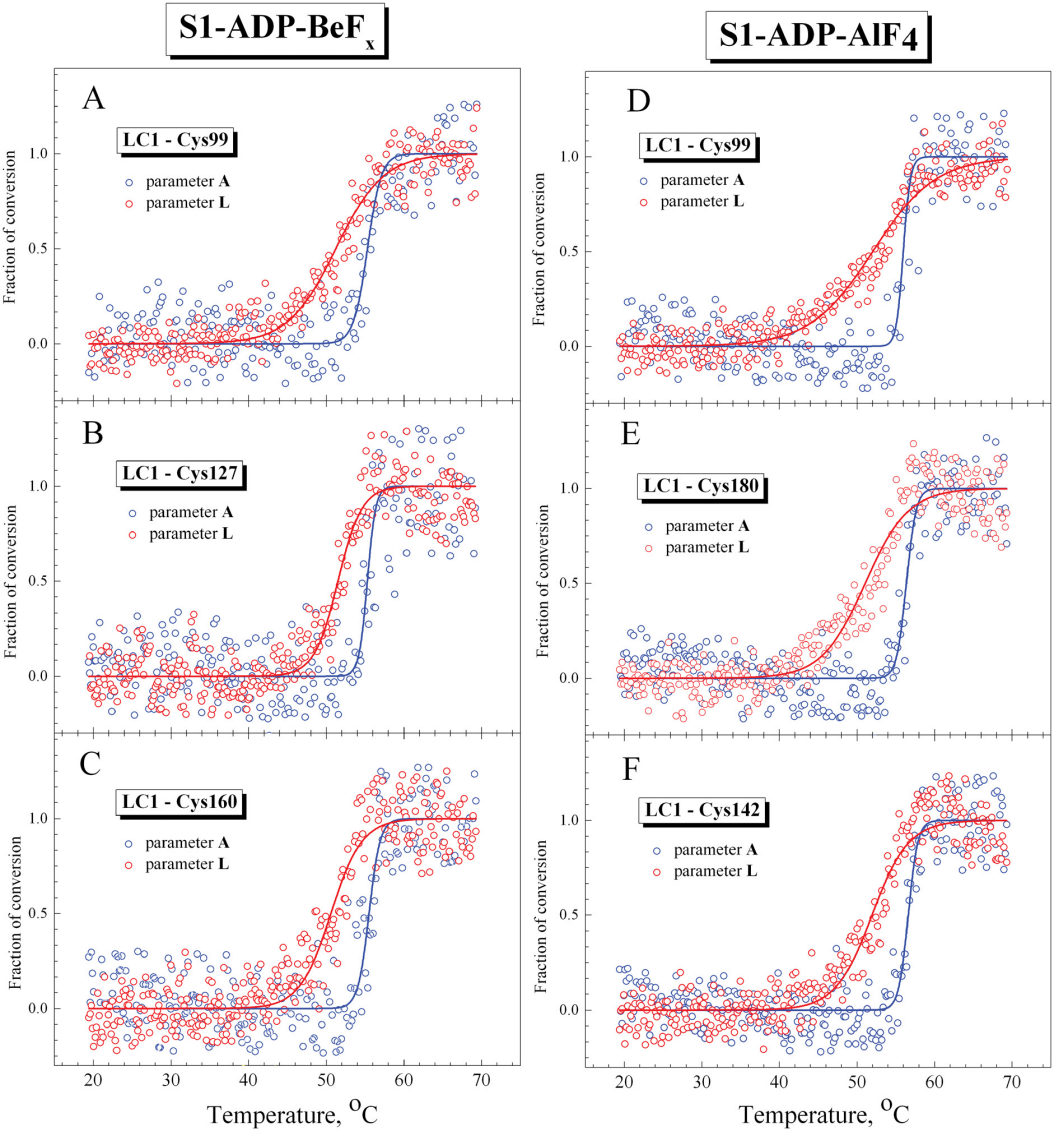
Representative temperature-induced changes in fluorescence measured on the S1 ternary complexes S1-ADP-BeF_x_ (A–C) and S1-ADP-AlF_4_ (D–F) for the labeled recombinant LC1 mutants associated with the S1 regulatory domain (red circles) and for the tryptophan fluorescence of S1 (blue circles). The AEDANS-labeled Cys residues in the LC1 were Cys-99 (A,D), Cys-127 (B), Cys-142 (F), Cys-160 (C), as well as Cys-180 of the wild-type LC1 (E). The changes in the fluorescence were measured by changes of the values of normalized parameters *L* and *A* for the labeled LC1 and tryptophan residues, respectively, which were calculated as described in Materials and methods. Red and blue solid lines represent the results of the data analysis according to a sigmoidal function (Boltzmann) for LC1 and tryptophan, respectively.

Fig 7 represents the temperature-induced changes in parameter *L* for fluorescence of the LC1 mutants associated with the regulatory domain of S1 in the ternary complexes S1-ADP-BeF_x_ and S1-ADP-AlF_4_, in comparison with the changes in parameter *A* for the tryptophan fluorescence, which reflect the thermal unfolding of the S1 motor domain in these complexes. The values of *T*_0.5_ and *d* extracted from the analysis of the temperature-induced changes in parameters *A* and *L* for all LC1 mutants studied are summarized in Table 3.

**Table 3:** The main parameters, *T*_0.5_ and *d*, obtained from the analysis of the temperature-induced changes in the fluorescent parameters *A* and *L* for tryptophan and AEDANS-labeled LC1 mutants, respectively. The data were obtained from experiments on the S1 ternary complexes S1-ADP-BeF_x_ and S1-ADP-AlF_4_ (Fig 7).

**Cys position in LC1**	**S1-Be-F_x_**			
	**parameter *A***		**parameter *L***	
	***T*_0.5_ (°C)**	***d***	***T*_0.5_ (°C)**	***d***
**Cys180**	56.0	1.14	52.8	1.9
**Cys160**	55.5	0.79	50.6	2.3
**Cys142**	54.5	1.3	49.5	3.0
**Cys127**	55.3	0.54	51.4	1.5
**Cys99**	55.4	1.13	51.4	3.3
**Cys position in LC1**	**S1-Al-F**_**4**_			
	**parameter *A***		**parameter *L***	
	***T***_**0.5**_ **(**°**C)**	***d***	***T***_**0.5**_ **(**°**C)**	***d***
**Cys180**	56.6	0.62	52.2	2.4
**Cys160**	56.0	0.31	51.2	2.1
**Cys142**	56.4	0.6	51.1	2.85
**Cys127**	56.8	0.9	51.8	1.7
**Cys99**	56.0	0.45	52.0	4.0

The absolute error of the given *T*_0.5_ values did not exceed ± 0.5°C for parameter *A* and ± 1.0°C for parameter *L*. The absolute error of the given *d* values did not exceed ± 0.2 for parameter *A* and ± 0.25 for parameter *L*.

The results presented in Fig 7 and Table 3 show that for all LC1 mutants studied the changes of parameter *L* occur at markedly lower temperature than the changes of parameter *A*. In all cases, the *T*_0.5_ values for parameter *L* are substantially lower, by 4–5°C, than those for parameter *A* (Table 3). Like in the case of the nucleotide-free S1 (Fig 5), in the case of the S1-ADP-BeF_x_ and S1-ADP-AlF_4_ complexes the slope of the curve for parameter *L* is more gentle than for parameter *A* (Fig 7) and therefore the *d* value for parameter *L* is much higher than this value for parameter *A* (sometimes even by a factor of 7–9 as in the case of the S1-ADP-AlF_4_ complexes with LC1 mutants containing Cys-160 or Cys-99) (Table 3). The width of the temperature range in which the changes in the fluorescent parameters occur is much broader for parameter *L* than for parameter *A* (Fig 7), and this is just the reason for the difference in the *d* values.

**Fig 8:**
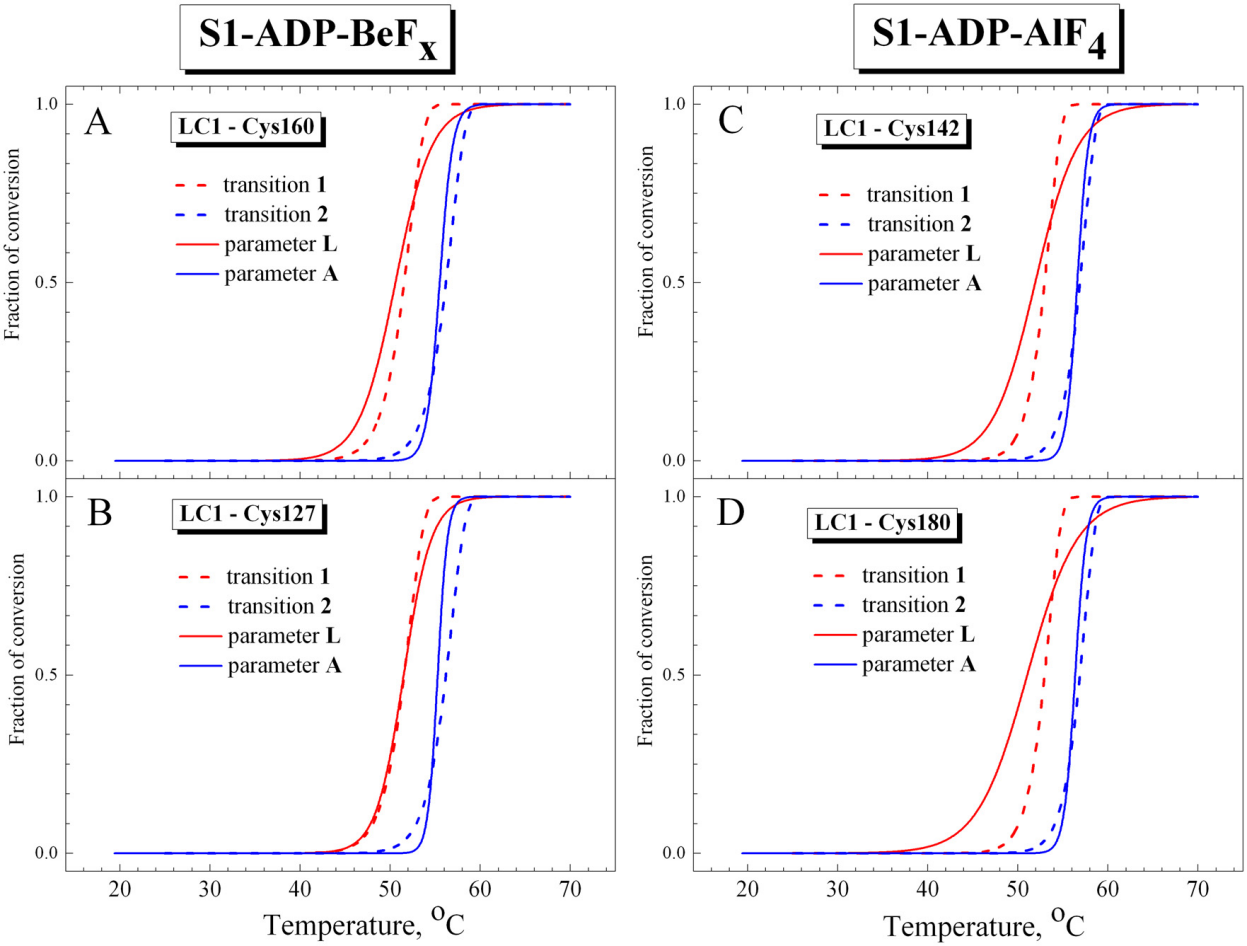
Representative temperature-induced changes in fluorescence for S1 in the complexes S1-ADP-BeF_x_ (A,B) and S1-ADP-AlF_4_^-^ (C,D) in comparison with fraction of conversion for the calorimetric transitions 1 and 2. The changes in the S1 fluorescence were measured by changes of the value of normalized parameter *A* for tryptophan residues and normalized parameter *L* for the AEDANS-labeled LC1 mutants associated with the S1 regulatory domain. Red and blue solid lines represent the results of the data analysis according to a sigmoidal function (Boltzmann) for LC1 and tryptophan, respectively. The labeled Cys residues in the LC1 were Cys-160 (A), Cys-127 (B) and Cys-142 (C), as well as Cys-180 of the wild-type LC1 (D). The curves corresponding to a fraction of conversion for the calorimetric transitions 1 and 2 (red and blue dashed lines, respectively) were taken from Fig 3.

Analyzing the *T*_0.5_ values for parameter *L* presented in Table 3, we can conclude that they are similar to the *T*_0.5_ values for calorimetric transition 1 obtained from the DSC experiments on the S1-ADP-BeF_x_ and S1-ADP-AlF_4_ complexes (Table 1). Moreover, we compared temperature dependences of normalized parameters *L* and *A*, which were obtained from the fluorescence experiments (Fig 7), with thermal transitions 1 and 2 obtained from DSC experiments on the S1-ADP-BeF_x_ and S1-ADP-AlF_4_ complexes and presented in Fig 3 as fractions of conversion from the native to the denatured state. The results of this comparison (Fig 8) demonstrate that the curves for parameter *A* almost fully coincide with calorimetric transition 2 corresponding to thermal denaturation of the S1 motor domain. In turn, the curves for parameter *L* are similar to calorimetric transition 1, although the changes of this parameter occur in wider temperature region than for transition 1 (Fig 8), probably due to significant dispersion of experimental data at the beginning and at the end of the heating (Fig 7). Nevertheless, a rather close similarity is observed between the calorimetric transition 1 and the temperature dependences of normalized parameter *L*, especially nearby the transition midpoint (Fig 8). Taking into account that the temperature-induced changes in parameter *L* most likely reflect structural changes that occur in LC1 (at least, in its C-terminal part) in the course of its thermal denaturation, we can conclude that calorimetric transition 1 observed on the DSC profiles of the S1-ADP-BeF_x_ and S1-ADP-AlF_4_ complexes corresponds to the thermal denaturation of the S1 regulatory domain containing associated LC1.

It should be noted that the temperature-induced changes in fluorescence of labeled LC1, which were observed in the S1-ADP-BeF_x_ and S1-ADP-AlF_4_ complexes, were similar for all LC1 mutants, independently of the position of a labeled cysteine residue in the C-terminal half of LC1 (Cys-99, Cys-127, Cys-142, Cys-160, or Cys-180) (Fig 7, Table 3). This was somewhat contrary to our expectations. We supposed that some of the labeled residues (e.g. Cys-142, Cys-160, and Cys-180), which are apparently located in the interface area between the C-terminal half of LC1 and the motor domain of S1 both in the absence of nucleotides and in the S1-ADP-BeF_*x*_ complex (Fig 1), should be more sensitive than other labeled residues to the presumable interdomain interaction. Correspondingly, we expected to reveal tangible difference in the thermally-induced changes of the fluorescence between these and other labeled residues. However, the results presented above (Figs 5 and 7, Tables 2 and 3) unequivocally indicate that LC1 (at least, its C-terminal half) undergoes thermally induced structural changes as a whole, and as a result, the changes in the microenvironment occur in a similar way for all labeled cysteine residues irrespective of their location relative to the interface area. We propose that dissociation of LC1 from the S1 heavy chain could potentially account for this phenomenon.

### Thermally induced dissociation of LC1 from the S1 heavy chain

**Fig 9:**
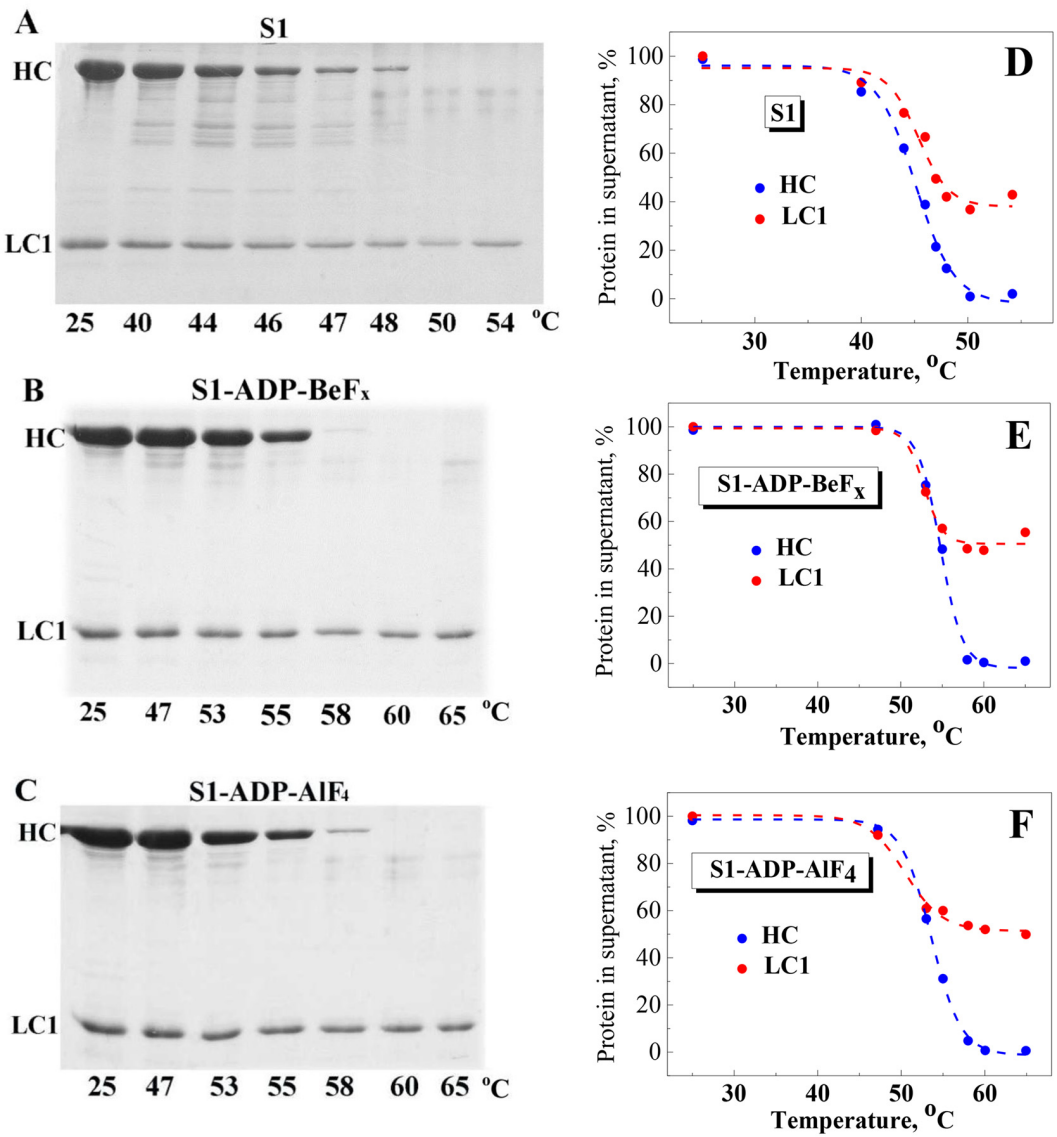
Temperature-induced dissociation of LC1 from the S1 heavy chain. The experiments were performed both on the nucleotide-free S1 (A) and on the S1 complexes S1-ADP-BeF_x_ (B) or S1-ADP-AlF_4_ (C). The samples were heated with a constant rate of 1°C/min; upon reaching the indicated temperature, aliquots (100 μL) were withdrawn, cooled down, subjected to ultracentrifugation, and the content of LC1 and the S1 heavy chain (HC) in the supernatants was determined by SDS–PAGE. Panels D, E, and F show quantification of the data presented in (A–C), i.e. disappearance of HC and LC1 from the supernatants during heating.

As described earlier and mentioned above, the irreversible thermal denaturation of S1 is usually accompanied by its aggregation (see Fig 6A), which is followed by precipitation of S1 expressed in a disappearance of the S1 heavy chain from supernatants obtained after ultracentrifugation of heated S1 [36]. It was also shown that ELCs dissociate from S1 upon heating and can be separated from aggregated and precipitated S1 heavy chains by an intensive ultracentrifugation [36]. Here we applied this approach to investigate the temperature-induced dissociation of LC1 from the S1 heavy chains in the S1-ADP-BeF_x_ and S1-ADP-AlF_4_ complexes. The samples were heated with a constant rate of 1°C/min and aliquots were withdrawn at appropriate temperature, cooled down, subjected to ultracentrifugation to separate LC1 from denatured and precipitated S1 heavy chains, and then protein composition of the supernatants was determined by SDS–PAGE. The results showed that the S1 heavy chain disappeared from the supernatants at temperatures corresponding to the thermal denaturation and aggregation of the S1 motor domain, i.e. above 40°C for the nucleotide-free S1 (Fig 9A and 9D) and above 50°C for the S1-ADP-BeF_x_, and S1-ADP-AlF_4_ complexes (Fig 9B, 9C, 9E and 9F). However, LC1 remained in the supernatant even when the S1 denaturation process was completed and the S1 heavy chain fully disappeared from the supernatants (at temperatures above 48°C for the nucleotide-free S1 and above 58°C for the S1-ADP-BeF_x_ and S1-ADP-AlF_4_ complexes). Under these conditions, about 40% of LC1 remained in the supernatant for the nucleotide-free S1 (Fig 9A and 9D) and more than 50% for the S1-ADP-BeF_x_ and S1-ADP-AlF_4_ complexes (Fig 9B, 9C, 9E and 9F). It should be noted that in all cases the LC1 content in the supernatants remained constant after full disappearance of the S1 heavy chains (Fig 9D–9F).

Partial disappearance of LC1 from the supernatants (Fig 9) can be explained as follows. We propose that all these light chains dissociate from the S1 heavy chains upon thermal unfolding of the S1 regulatory domain. However, some of them, either free in solution or bound to regulatory domains of native or only partially denatured S1 molecules, can be mechanically involved into amorphous aggregates of the S1 heavy chains. This process can only take place until the complete denaturation of all S1 molecules. This may explain why LC1 disappears from the supernatants within the temperature range of S1 thermal unfolding (up to 48°C for the nucleotide-free S1 and up to 58°C for the S1-ADP-BeF_x_ and S1-ADP-AlF_4_ complexes), whereas its content in the supernatant remains constant after complete denaturation of all S1 molecules, when all denatured S1 heavy chains precipitate and disappear from the supernatants (Fig 9).

Collectively, the results presented above show that thermal unfolding of S1 is accompanied by dissociation of LC1 from the S1 heavy chain not only in the absence of nucleotides, but also in the complexes S1-ADP-BeF_x_ and S1-ADP-AlF_4_. This dissociation leads to a shift of the fluorescence spectrum of labeled LC1 towards shorter wavelengths, as was shown by comparison of the spectra obtained for LC1 free in solution and bound to the S1 regulatory domain (see S1 Fig). This spectral shift can explain the increase in parameter *L* observed for fluorescently labeled LC1 upon thermal unfolding of S1, which is accompanied by dissociation of LC1 from the S1 heavy chain. This dissociation in turn can explain why the changes in parameter *L* occur in a similar way for all labeled cysteine residues irrespective of their location relative to the interface area (Fig 7). Probably the whole LC1 (at least, its C-terminal half) denatures and immediately dissociates from the S1 heavy chain resulting in similar changes in parameter *L* for all fluorescent probes located in this region.

## Discussion

The data presented here show that the formation of the ternary complexes S1-ADP-AlF_4_^-^ and S1-ADP-BeF_x_ which mimic the S1 ATPase intermediate states S1**-ADP-P_i_ and S1*-ATP, respectively, significantly stabilize not only the motor domain, but also the regulatory domain in the S1 molecule. Comparing the thermally-induced changes that occur in the fluorescence of labeled LC1 bound to the S1 regulatory domain in these complexes with the corresponding changes in the nucleotide-free S1, we can conclude that formation of these complexes leads to a significant increase in the *T*_0.5_ value for parameter *L*, from 45–46°C (Table 2) to 50–52°C (Table 3). This means that LC1 associated with the S1 regulatory domain in the S1-ADP-AlF_4_^-^ and S1-ADP-BeF_x_ complexes denatures at higher temperature than in the nucleotide-free S1 (see Fig 1). This increase in thermal stability of LC1 probably reflects stabilization of the entire regulatory domain. One can propose the following possible explanations for this stabilization. First is that the global structural changes in the motor domain, which occur upon ATP binding and hydrolysis, not only lead to rotation of the converter, but also stabilize it, and this stabilization is transmitted through the pliant region to the α-helix of regulatory domain and to LC1 associated with this helix. However, it seems unlikely that conformational changes in converter can be transmitted for such a long distance to the entire regulatory domain. Another explanation for this stabilization, which is consistent with previous findings [9–12], is the nucleotide-induced interaction of the S1 regulatory domain (more precisely, LC1 bound to the regulatory domain) with the motor domain due to rotation of the lever arm relative to the motor domain. In general, both these explanations are not contradictory and rather mutually complement each other.

Our results also demonstrate a strong stabilization of the S1 motor domain in the S1-ADP-AlF_4_^-^ and S1-ADP-BeF_x_ complexes whose formation leads to a significant increase in the *T*_0.5_ value for parameter *A* of tryptophan fluorescence, from 46–47°C for the nucleotide-free S1 (Table 2) up to 55–57°C in the complexes (Table 3). This is consistent with the previous DSC data that the thermal stability of the isolated motor domain of *Dictyostelium discoideum* myosin II (M765) strongly increases upon formation of its ternary complexes with ADP and V_i_, BeF_x_ or AlF_4_ [40, 41]. Significant stabilization, by ~ 9°C, of the motor domain in the S1 molecule also follows from the comparison between calorimetric transitions assigned to this domain in the nucleotide-free S1 [28] (Table 1) and in the complexes S1-ADP-AlF_4_^-^ or S1-ADP-BeF_x_ (Figs 2 and 3, Table 1). Noteworthy, in all these cases a close similarity was observed between the transitions obtained from DSC and fluorescence experiments (e.g., see Figs 3 and 8).

The situation is more complicated in the case of the S1 regulatory domain for which a similarity between the DSC and fluorescence data was clearly observed only for the S1-ADP-AlF_4_^-^ and S1-ADP-BeF_x_ complexes (Fig 8), but not for the nucleotide-free S1. In the latter case, for all LC1 mutants studied the changes in fluorescence of labeled LC1 were observed at much higher temperature (*T*_0.5_ = 45–46°C, Table 2) than it was predicted from previous DSC experiments [28] for the thermal denaturation of the regulatory domain in the nucleotide-free S1 (i.e. ~40°C). Moreover, no appreciable changes in the fluorescence of labeled LC1 were observed at 40°C (Fig 5). We can propose the following possible explanations for this contradiction between the results obtained from DSC and fluorescence experiments.

First, it should be noted that all fluorescently labeled cysteine residues were located in the C-terminal half of LC1, which was predicted to interact with the S1 motor domain [8, 9, 12, 13] (see Fig 1). We cannot exclude a possibility that the N-terminal lobe of LC1, which does not contain fluorescently labeled cysteines, denatures upon heating at lower temperature (e.g. at about 40°C) than the C-terminal lobe, thus demonstrating the least thermostable calorimetric domain 1 with *T*_0.5_ at ~40°C on the DSC profile. This is consistent with the data of crystallographic studies which demonstrated that the N- and C-terminal lobes of ELC are characterized by different conformations when bound to the S1 heavy chain [2, 42]: the N-terminal lobe is in a “closed” conformation, whereas the C-terminal lobe is characterized by a particular “semi-open” conformation which provides more tight binding to the heavy chain of the S1 regulatory domain. On the other hand, it seems likely that thermal unfolding of the N-terminal half of LC1is not accompanied by dissociation of LC1 from the S1 heavy chain and, correspondingly, by the changes in fluorescence of labeled Cys residues located in the LC1 C-terminal half. This may explain, at least partly, the above mentioned contradiction between the DSC and fluorescence results obtained on the nucleotide-free S1.

Another possible explanation for this contradiction follows from the recently published data obtained from negative-stain electron microscopy and image processing studies on the nucleotide-free S1, which showed a marked flexibility of S1 reflected in variation in the orientation of the S1 regulatory domain (lever arm) relative to the motor domain, i.e. in the angle between these domains, with a hinge point located in the pliant region between the converter in the motor domain and the ELC in the regulatory domain [43]. We cannot exclude a possibility that these changes in the orientation of the S1 regulatory domain may be accompanied by additional contacts of the C-terminal half of LC1 with the S1 motor domain, which, in turn, may lead to an increase in the temperature of the changes in fluorescence of the labeled LC1.

In any case, we conclude that the results obtained from our experiments on the thermal unfolding of S1 testify in favor of the earlier proposed concept of the nucleotide-induced interaction between the S1 motor domain and LC1 associated with the S1 regulatory domain. Using fluorescently labeled LC1s, we have identified the least thermostable thermal transition on the DSC profiles of the S1-ADP-AlF_4_^-^ and S1-ADP-BeF_x_ complexes as the regulatory domain of the S1 molecule and also have shown that formation of these complexes (stable analogs of the S1 ATPase intermediate states S1**-ADP-P_i_ and S1*-ATP) strongly increases the thermal stability of the S1 regulatory domain. We propose that this stabilization can be explained not only by global nucleotide-induced conformational changes of the S1 molecule leading to rotation of the converter and the lever arm, but also by a rather tight interaction of LC1 on the regulatory domain with the motor domain, which is caused by this rotation. The interaction of the C-terminal part of LC1 with the S1 motor domain can also exist in the absence of nucleotides [1, 8] (Fig 1A). However, our results indicate that this interaction becomes much tighter in the S1-ADP-AlF_4_^-^ and S1-ADP-BeF_x_ complexes that mimic the main intermediate states of the S1 ATPase cycle, in which the C-terminal part of LC1 interacts with the other sites on the motor domain [9] (Fig 1B). This is consistent with the previous findings which showed that binding of ATP to the active site on the motor domain leads to a significant decrease in the mobility of the C-terminal part of LC1 [12].

According to crystallographic data, formation of the S1-ADP-AlF_4_^-^ and S1-ADP-BeF_x_ complexes leads to a significant movement of the regulatory domain (lever arm) relative to the motor domain, and as a result, in these complexes the lever arm is located rather close to the surface of the motor domain [9] (Fig 1B). The results presented in the current work indicate that in these complexes not only the regulatory domain and the motor domain are located close to each other due to rotation of the regulatory domain, but tight interaction may occur between both these domains of the myosin head. These results suggest that during the ATPase cycle the myosin head undergoes global changes in its domain structure, which are expressed in the tight coupling between the two main parts of the head, the motor domain and the regulatory domain. It seems likely that this interaction between the two domains, which probably occurs during the ATPase cycle, may play a rather important role in functioning of the myosin head as a molecular motor. For instance, the immobilization of the lever arm on the surface of the motor domain may be important for stabilization of the pre-power stroke state of the myosin head in the course of the ATPase cycle.

## Supporting Information

S1 Fig Comparison of normalized fluorescence spectra obtained for labeled LC1 free in solution (red line) and bound to the S1 regulatory domain (dashed blue line). The value of λ_max_ was equal to 491 nm for LC1 bound to the S1 heavy chain and 486.4 nm for free LC1.
(TIF)
