# Supplementary figures and images for: Does Interaction between the Motor and Regulatory Domains of the Myosin Head Occur during ATPase Cycle? Evidence from Thermal Unfolding Studies on Myosin Subfragment 1
Source: PLoS One. 2015 Sep 10;10(9):e0137517. doi: 10.1371/journal.pone.0137517 (PMC4565648; doi:10.1371/journal.pone.0137517)

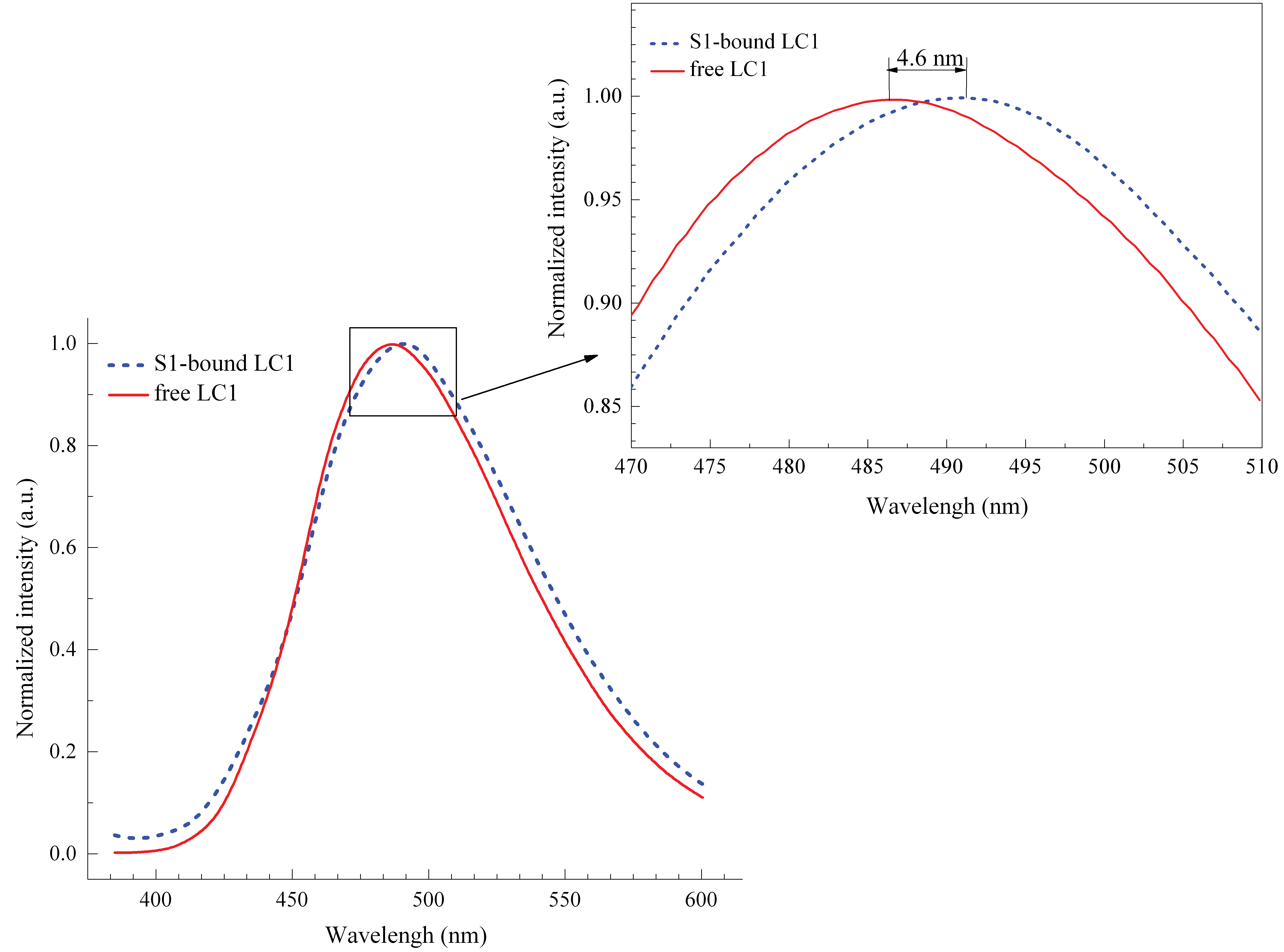

Supplement: S1 Fig — The value of λmax was equal to 491 nm for LC1 bound to the S1 heavy chain and 486.4 nm for free LC1. (TIF) [file pone.0137517.s001.tif]
